# Impact of air pollution on pulmonary function and respiratory symptoms in children. Longitudinal repeated-measures study

**DOI:** 10.1186/1471-2466-10-62

**Published:** 2010-11-24

**Authors:** Benigno Linares, Juan M Guizar, Norma Amador, Alfonso Garcia, Victor Miranda, Jose R Perez, Rocío Chapela

**Affiliations:** 1 Unidad Médica de Alta Especialidad No. 1, Instituto Mexicano del Seguro Social. López Mateos e Insurgentes s/n Col. Paraísos Z.C. 37320. León, México; 2 Departamento de Medicina y Nutrición, Universidad de Guanajuato. 20 de Enero 929 Col. Obregón Z.C. 37320. León, México; 3 Instituto Nacional de Salud Pública, Cuernavaca, Morelos, México; 4 Instituto Nacional de Enfermedades Respiratorias, Tlalpan 4502, México DF; 5 Universidad De La Salle Bajío, León Gto, México

## Abstract

**Background:**

Salamanca, Mexico occupied fourth place nationally in contaminating emissions. The aim of the study was to determine the impact of air pollution on the frequency of pulmonary function alterations and respiratory symptoms in school-age children in a longitudinal repeated-measures study.

**Methods:**

We recruited a cohort of 464 children from 6 to 14 years of age, from two schools differing in distance from the major stationary air pollution sources. Spirometry, respiratory symptoms and air pollutants (O_3, _SO_2_, NO, NO_2_, NOx, PM_10_,) were obtained for each season. Mixed models for continuous variables and multilevel logistic regression for respiratory symptoms were fitted taking into account seasonal variations in health effects according to air pollution levels.

**Results:**

Abnormalities in lung function and frequency of respiratory symptoms were higher in the school closer to major stationary air pollution sources than in the distant school. However, in winter differences on health disappeared. The principal alteration in lung function was the obstructive type, which frequency was greater in those students with greater exposure (10.4% vs. 5.3%; OR = 1.95, 95% CI 1.0-3.7), followed by the mixed pattern also more frequent in the same students (4.1% vs. 0.9%; OR = 4.69, 95% CI, 1.0-21.1). PM_10 _levels were the most consistent factor with a negative relationship with FVC, FEV_1 _and PEF but with a positive relationship with FEV_1_/FVC coefficient according to its change per 3-month period.

**Conclusions:**

Students from the school closer to major stationary air pollution sources had in general more respiratory symptoms than those from the distant school. However, in winter air pollution was generalized in this city and differences in health disappeared. PM_10 _levels were the most consistent factor related to pulmonary function according, to its change per 3-month period.

## Background

Air pollution has been associated to several adverse health effects that depend on the physical and chemical properties of contaminants, time and frequency of exposure. However, most information is about acute health effects of air pollution, but health effects due to chronic exposure are not as well known. Since 1994, Mexico has been ranked by the World Health Organization as a country with serious environmental pollution problems generated by industry and vehicles. It is estimated that, for this year, the contaminating industrial emissions in urban areas were close to 1,965,965 tons [1]. The city of Salamanca, Mexico occupies fourth place nationally in contaminating emissions. For example, the concentrations of ozone (O_3_), sulfur dioxide (SO_2_), and particulate matter (PM_10_) exceeded the levels allowed by the Mexican official norm for up to 70 days every year since 2002 to 2004. Furthermore, this municipality first place in mortality and the second in respiratory infections for the entire state in this period [2]. Acute respiratory tract infections (ARTI) in the group between 5 and 14 years of age have been the principal cause of morbidity in outpatient consults, and asthma occupied third place in consults [2]. Nevertheless, it is not known if the impact from contamination varies with the different seasons. The purpose of this study was to determine the impact of air pollution on the frequency of pulmonary function alterations and respiratory symptoms in school-age children in a longitudinal repeated-measures study.

## Methods

**Figure 1:**
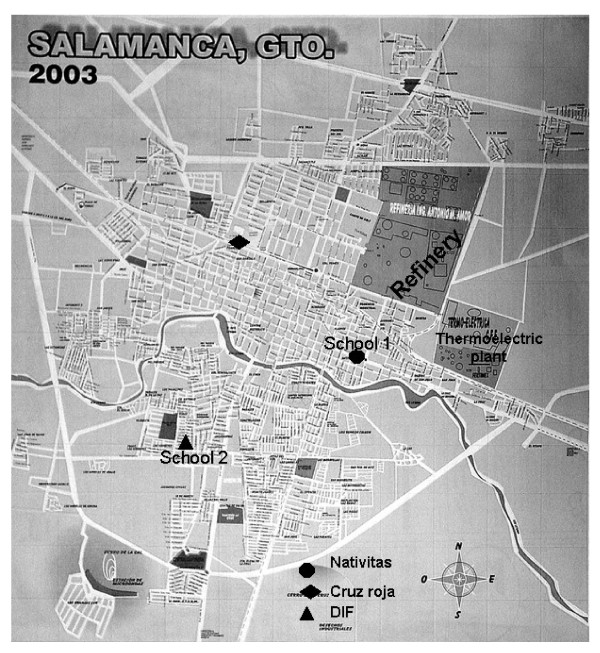
**Map describing schools position according to the principal sources of air pollution**.

The study took place in the urban area of Salamanca, Mexico from March, 2004 to February, 2005. The municipality has a territorial extension of 774,000 km^2 ^(2.53% of the total state surface) and a population of approximately 226,454 inhabitants. Its climate is temperate most the time, and it forms part of the industrial corridor of the state. Supported by air quality reports during 2005, the city was divided in three zones according to the O_3 _levels and to the number of days that exceeded the air quality allowed by the Mexican official norm. The main sources of air pollution in Salamanca are the oil refinery and the thermoelectric plant. The average ozone levels in the year 2005 were 0.08, 0.04 and 0.02 ppm for zones I, II and III, respectively, and the number of days that exceeded the norm for each of the zones were 50, 2 and 0, respectively. A primary school closer to major stationary air pollution source (school 1) and another in the distant area (school 2) were randomly selected. School 1 was 1100 m from the petrochemical industrial zone (oil refinery and thermoelectric plant), whereas school 2 was 7300 m away (Figure 1).

Required sample size was estimated as 434 participants to detect a difference of 15% in frequency of respiratory symptoms between the 2 schools studied, according to comparison for proportions [3], and we added a 15% for drop outs (500 children). The hoped difference of 15% between schools was obtained from a previous frequency reported in Latin children [4]. All children were randomly selected (250 for each school) from the total student registry providing the students living and attending the school in the corresponding zone for at least the previous 3 years. Students with cardiopathies, cleft lip or palate, facial paralysis or any other alteration that would prevent spirometry from being performed were excluded. Permission was obtained from the authorities of the State Department of Education and from the participating schools. The parents, as well as each of the participants, were informed about the objective of the study and the procedures before obtaining consent to participate. Approval for the study was granted by the local Investigation Committee of the Mexican Institute of Social Security. Clinical and demographic history was obtained through the questionnaire proposed by the International Study of Asthma and Allergies in Childhood (ISAAC), validated for the Spanish language [4]. Use of fossil full (including charcoal and woodcutter) frequently used in Mexico was considered. The baseline survey occurred in winter. Children ≤ 9 years and their parents answered together, whereas older children answered for themselves. Questions were asked about respiratory symptoms (wheezing, rhinorrhea, eczema, respiratory infection, dyspnea and hospitalization secondary to acute respiratory infection). Atopy was considered in case of previous diagnosis of asthma, allergic rhinitis or atopic dermatitis. We performed anthropometric measurements to obtain height, weight, and body mass index (BMI). Socioeconomic level was estimated using family income and considering the level of education of parents and number of rooms in the house.

Technicians were trained specifically for this project, using the guidelines of the Spirometry Course of the National Institute of Occupational Safety and Health in the U.S. Spirometries were accomplished with the EasyOne spirometer (NDD, Technopark, Zurich Switzerland) that meets the quality criteria established by the American Thoracic Society (ATS), 1994 [5]. Each student underwent a forced spirometry to obtain the following parameters: forced vital capacity (FVC), forced expiratory volume in the first second (FEV_1_), relation of FEV_1_%/FVC and peak expiratory flow (PEF). The quality of spirometric tests was assessed by several criteria in addition to the automatic evaluation done by the software device. One was the number of acceptable maneuvers according to ATS, 1 ranging from 0 to 3, the highest kept by the spirometric software. Another indicator of quality was reproducibility. FEV_1 _and FVC were considered reproducible according to ATS criteria when the best two trials differed by not more than 200 mL. A total of 97.5% of the tests achieved reproducibility within 150 mL fulfilling the 2005 ATS-ERS criteria. Reference values of Hankinson et al. for Mexican-Americans were used [6], considering that children > 7 years old can fulfill ATS criteria of quality after the first spirometric evaluation [7]. The presence of spirometric values below the 5^th ^percentile of reference values were considered abnormal. The obstructive pattern was defined by the diminution of the FEV_1 _and FEV_1_/FVC index, the restrictive pattern by diminution of the FVC, with normal FEV_1_/FVC index, and mixed pattern by diminution of FVC and FEV_1_.

Each participant was interviewed and underwent to a forced spirometry during each season of the year in a study panel of four measurements per subject, taken at the beginning (day 15 to 35) of each season.

The data of the environmental conditions and aerial polluting agents were provided by the network of atmospheric monitoring of Salamanca. This system has three fixed stations of monitoring distributed in strategically important points of the city; figure 1 shows the location of the three fixed stations of environmental monitoring. The registration of main gaseous pollutants of the air was obtained from the automated environmental monitoring of the Institute of Ecology of the State of Guanajuato that includes the approval of the Environmental Protection Agency (EPA). O_3 _levels were measured by UV spectrophotometry (Thermo Environmental gas analyzer, model 49 C); SO_2 _by UV fluorescence (Thermo Environmental gas analyzer, model 43 C); NO_2 _by chemical luminescence (Thermo Environmental gas analyzer, model 42 C), CO by non-dispersive infrared (NDIR) technique (Thermo Environmental gas analyzer, model 48 C) and PM_10 _by using the tapered element oscillating microbalance (TEOM).

The quality of this information is regularly assessed and assured by the National Institute of Ecology. All the stations measured daily O_3, _SO_2_, carbon monoxide (CO), nitrogen monoxide (NO), nitrogen dioxide (NO_2_), total oxides of nitrogen (NOx), and PM_10_.

### Statistical analysis

Baseline characteristics between the two study groups were compared by a Student t-test for independent groups (continuous variables) and by chi squared test (categorical variables). Children's air pollutant exposure was calculated based on air pollutant levels at the closest fixed-site monitor to his/her school. Schools were located within 2 kilometers from one of the 3 selected fixed-site air pollution monitoring stations. We obtained the 8-hr maximum moving average for O_3_; we also obtained the 24 hour mean for SO_2 _and PM_10 _transformed to only taking into account the days with valid information for more than 75% of the hours. Days with non-valid information were assigned as missing data. In this analysis, exposure to CO, NO and NO_2 _showed the low levels registered.

To evaluate the symptom data we used multilevel logistic models, clustering by child and using the intercept as a random effect. In addition, adaptive quadrature with Newton Raphson iterations was used instead of ordinary quadrature. Different choices of integration points were made as long as they showed a more adequate and superior model fit, however results did not vary significantly. Symptoms coded as dummy variables played the role of outcome variables. To analyze the association between lung function parameters and air pollutants, we used mixed models with the same characteristics previously described, adjusting for potential confounding factors such as height, BMI, sex, age, fossil fuel and passive smoking.

To estimate deficits in lung function, generalized linear mixed models were fitted. We examined random intercept models, including both, age and sex as fixed effects, against random intercepts and slopes models, including age, sex, and the interaction terms age*pollutant*sex; and age*pollutant as fixed effects and age as a random effect.

For the three lung function parameters, FVC, FEV_1 _and PEF (outcome variables), we obtained the best goodness-of-fit statistics, smaller Akaike's Information Criterion (AIC), and a variance of the random slope not equal to zero. A statistically significant interaction term between age and sex was obtained as well, which indicates the three lung function parameters (FVC, FEV_1_, and PEF) intercept, and rate of change over age and sex.

We also considered a three-level model design as follows: first level models included time-specific covariates such as age, previous day mean for the same air pollutant, body mass index, body mass index squared, and residuals of the regression height on age. The second stage models included monitoring stations - specific covariates. In this stage, we considered the mean average pollutant level over the previous year assigned to every subject on each visit. The third level models included subject - specific covariates; the mean average pollutant level per monitor over the study period was fitted on this stage. These models were also adjusted for potential confounding factors.

For statistical analysis, we used Stata 9.0 (Stata Corporation, College Station, Texas, EUA) and the GLLAMMs program (Generalized Linear Latent and Mixed Models) [8]. PROC MIXED in SAS (Version9) was used to fit mixed models and to estimate deficits in lung function.

## Results

### Population and Exposure to Contaminants

We studied 464 participants aged 6 to 14 years. Two hundred and thirty nine (51.5%) belonged to school 1 and 225 (48.5%) to school 2. Both schools were public, and socioeconomic level in all participants was similar. We had 35 drop outs during the study (5 decided not continue with the study, and 30 changed school during the study). Table 1 shows the characteristics of the population studied. The first from 5 evaluations (baseline, spring, summer, fall and winter) was performed on winter and no significant difference in age, gender, BMI and lung function tests were found between groups. However, ARTI were more frequent in those children from school 1.

**Table 1:** Baseline characteristics of children according to school, Salamanca, 2004 - 2005

	School 1 n = 239	School 2 n = 225	p
Gender (female/male)	118/121	117/108	0.60
Age (years)	9.0 ± 1.6	9.2 ± 1.5	0.11
Weight (k)	34.8 ± 11.4	36.4 ± 11.6	0.13
Height (cm)	134.0 ± 10.8	135.5 ± 10.6	0.08
BMI	19.1 ± 3.7	19.1 ± 3.6	0.75
Previous diseases and symptoms n (%)			
Bronchitis	29 (12.1)	24 (10.6)	0.74
Pneumonia	17 (7.1)	9 (4.0)	0.14
Cough	219 (91.6)	207 (86.6)	0.16
Acute asthma	3 (1.2)	0 (0.0)	0.34
Acute respiratory tract infections	221 (92.4)	178 (79.1)	0.001
Hospitalization for asthma	2 (0.8)	0 (0.0)	0.60
Hospitalization for acute respiratory tract infections	13 (5.4)	5 (2.0)	0.07
FEV_1 _(%p)*	102.5 ± 21.1	101.5 ± 14.4	0.55
FVC (%p)*	109.3 ± 23.7	109.2 ± 18.0	0.95
FEV_1_/FVC (c)	92.6 ± 9.8	91.6 ± 10.0	0.24
PEFR (%p)	106.5 ± 26.7	107.1 ± 24.3	0.81

* Expressed spirometric values such as percentage of the predicted value according to reference values for Mexican children

Air pollutant in general showed higher levels in school 1 during spring and summer, however this pattern was inverse in fall and winter when all pollutants increased significantly in school 2 (table 2).

**Table 2:** Pollutant levels and climatologically data in the studied zones during all seasons; Salamanca, Guanajuato, 2004 - 2005

Variables		*Spring*	*Summer*	*Fall*	*Winter*
O_3 _(μg/m^3^)	School 1	28.7 ± 2.7	28.1 ± 0.7	15.4 ± 0.1	20.2 ± 0.7
	School 2	17.6 ± 0.3	23.6 ± 1.6	39.8 ± 1.3	33.2 ± 1.9
	p*	0.001	0.001	0.001	0.001
SO_2 _(μg/m^3^)	School 1	31.2 ± 1.1	32.1 ± 0.7	27.2 ± 0.7	25.8 ± 0.4
	School 2	23.9 ± 1.1	23.0 ± 1.4	26.8 ± 1.6	35.6 ± 1.9
	p*	0.002	0.001	0.01	0.001
CO (ppm)	School 1	0.84 ± 0.09	0.37 ± 0.07	0.26 ± 0.01	0.81 ± 0.09
	School 2	1.4 ± 0.4	1.9 ± 0.9	2.4 ± 1.4	5.8 ± 0.9
	p*	0.001	0.001	0.001	0.001
NO (μg/m^3^)	School 1	18.4 ± 1.9	11.1 ± 0.4	4.7 ± 1.9	15.6 ± 1.3
	School 2	18.5 ± 1.7	6.6 ± 1.1	126.0 ± 7.7	65.4 ± 15.1
	p*	0.75	0.015	0.001	0.001
NO_2 _(μg/m^3^)	School 1	66.4 ± 4.2	12.4 ± 0.2	7.4 ± 0.2	14.9 ± 1.9
	School 2	6.3 ± 1.5	2.6 ± 0.6	51.6 ± 17.1	28.7 ± 3.0
	p*	0.001	0.024	0.001	0.002
NOx (μg/m^3^)	School 1	81.6 ± 6.9	19.6 ± 0.5	11.5 ± 0.2	27.8 ± 2.0
	School 2	24.6 ± 2.5	6.9 ± 1.3	156.1 ± 9.3	92.2 ± 21.4
	p*	0.001	0.018	0.001	0.001
PM_10 _(μg/m^3^)	School 1	77.9 ± 2.9	60.7 ± 1.4	41.7 ± 1.0	60.8 ± 3.3
	School 2	53.1 ± 24.4	11.3 ± 3.2	7.4 ± 0.7	77.9 ± 2.9
	p*	0.001	0.001	0.013	0.001
Temperature (°C)	School 1	19.3 ± 0.1	20.9 ± 0.1	20.2 ± 0.4	16.2 ± 0.7
	School 2	15.4 ± 0.5	16.8 ± 0.1	16.5 ± 0.1	12.1 ± 0.5
	p*	0.001	0.028	0.007	0.001
Relative humidity (%)	School 1	54.0 ± 0.9	52.9 ± 0.7	74.6 ± 0.1	65.7 ± 0.6
	School 2	50.9 ± 1.2	50.4 ± 2.3	74.0 ± 0.8	66.4 ± 2.5
	p*	0.02	0.078	0.129	0.001

*p value for differences between schools

### Allergic Diseases and Respiratory Symptoms

According to the ISAAC questionnaire, the total frequency of allergic diseases during the year was greater in students from school 1 than those from school 2: asthma (22.5% vs. 9.7%; p = 0.0005); rhinitis (44.7% vs. 34.2% p *= *0.01); and eczema (13.3% vs. 4.4%; p = 0.001).

According to the different seasons, the frequency of wheezing, rhinorrhea and dyspnea was higher in school 1 than in school 2 during spring. In summer, only wheezing was higher in this school, whereas rhinorrhea was higher in fall without differences in the rest of the year. ARTI was more frequent in the same school during fall and winter, whereas the hospitalization secondary to ARTI was only significantly higher in school 1 than in school 2 in winter (Table 3).

**Table 3:** Frequency of respiratory symptoms by school during the different seasons of the year; Salamanca, Guanajuato, 2004 - 2005

Variables		Spring	Summer	Fall	Winter
Wheezing n (%)	School 1	50 (20.9)	25 (10.4)	25 (10.4)	220 (92.0)
	School 2	22 (9.7)	12 (5.3)	16 (7.1)	216 (96.0)
	p*	0.0001	0.04	0.20	0.61
Rhinorrhea n (%)	School 1	139 (58.1)	82 (34.3)	84 (35.1)	74 (30.9)
	School 2	88 (39.1)	66 (29.3)	67 (29.7)	61 (27.1)
	p*	0.0001	0.25	0.03	0.36
Eczema n (%)	School 1	12 (5.0)	18 (7.5)	6 (2.3)	31 (12.9)
	School 2	11 (4.8)	11 (4.8)	11 (4.8)	10 (4.4)
	p*	0.94	0.40	0.17	0.001
Acute respiratory tract infection n (%)	School 1	78 (32.6)	54 (22.5)	38 (15.8)	206 (86.1)
	School 2	57 (25.3)	43 (19.1)	77 (34.2)	178 (79.1)
	p*	0.50	0.35	0.001	0.04
Dyspnea n (%)	School 1	44 (18.4)	26 (10.8)	17 (7.1)	36 (15.0)
	School 2	25 (11.1)	20 (8.8)	21 (9.3)	26 (11.5)
	p*	0.02	0.47	0.38	0.26
Hospitalization for ARTI n (%)	School 1	2 (0.8)	2 (0.8)	2 (0.8)	17 (7.1)
	School 2	1 (0.4)	1 1 (0.4)	1 1 (0.4)	5 (2.2)
	p*	0.59	0.59	0.59	0.01

*p value for differences between schools

Multilevel logistic models showed significant associations of O_3_, PM_10 _and SO_2 _with respiratory symptoms. An increase of 10 μg/m^3 ^in O_3 _ambient levels was associated with a significant increase in wheezing (OR = 1.0460), rhinorrhea (OR = 1.0429), ARTI (OR = 1.0820) and dyspnea (OR = 1.0313). Furthermore, significant increase in frequency of symptoms in children, associated with a 10 μg/m^3 ^increase in PM_10 _levels were found in wheezing (OR = 1.0478), rhinorrhea (OR = 1.0318), ARTI (OR = 1.0977), and dyspnea (OR = 1.0184). In addition, odds ratio for wheezing and ARTI were 1.0213 and 1.0521 respectively, for each 10 μg/m^3 ^increase in SO_2 _ambient levels. No significant effects were observed with NO_2 _(Table 4).

**Table 4:** Association between respiratory symptoms and air pollutant levels and other exposures sources among infant residents in Salamanca, Guanajuato 2004 - 2005

Respiratory symptoms						
	Wheezing OR^† ^(95%CI)	Rhinorrhea OR^† ^(95%CI)	Eczema OR^† ^(95%CI)	ARTI OR^† ^(95%CI)	Dyspnea OR ^† ^(95%CI)	Hospitalization by ARTI OR^† ^(95%CI)
**O**_**3 **_**(***μg/m*^3^**)**	1.0464 (1.0160, 1.0777)*	1.0429 (1.0000, 1.0881)*	0.9973 (0.9770, 1.0181)	1.0820 (1.0405, 1.1251)*	1.0313 (1.0025, 1.0609)*	1.0008 (0.9893, 1.0123)
**SO**_**2 **_**(***μg/m*^3^**)**	1.0213 (1.0018, 1.0413)*	0.9929 (0.9675, 1.0209)	0.9969 (0.9835, 1.0105)	1.0521 (1.0254, 1.0794)*	1.0082 (0.9893, 1.0271)	0.9985 (0.9910, 1.0061)
**NO**_**2 **_**(***μg/m*^3^**)**	0.8432 (0.7630, 1.0318)	0.8443 (0.7312, 1.0748)	1.0714 (0.9990, 1.1490)	0.8942 (0.7832, 1.0208)	0.9236 (0.8394, 1.0167)	1.0378 (0.9982, 1.0791)
**PM**_**10 **_**(***μg/m*^3^**)**	1.0478 (1.0329, 1.0630)*	1.0318 (1.0107, 1.0534)*	0.9974 (0.9875, 1.0075)	1.0977 (1.0771, 1.1188)*	1.0184 (1.0044, 1.0378)*	0.9991 (0.9935, 1.0047)
**Temperature (°C)**	0.6520 (0.5407, 0.7863)*	0.8914 (0.6810, 1.1668)	1.0457 (0.9174, 1.0192)	0.3842 (0.2998, 2.0312)	0.8669 (0.7242, 1.0378)	0.9975 (0.9273, 1.0730)
**School**	1.6696 (1.2966, 2.499)*	1.5749 (1.0955, 2.2645)*	0.8596 (0.7206, 1.0253)	1.5218 (1.0897, 2.1252)*	1.2422 (0.9745, 1.5834)	0.9386 (0.8508, 1.0355)
**Atopy**	1.0535 (0.9735, 1.1400)	1.1192 (1.0102, 1.2399)*	1.0493 (1.0018, 1.0991)*	1.0873 (0.9973, 1.1855)	1.0631 (0.9901, 1.1415)	1.0257 (0.9996, 1.0525)
**Allergen**	1.0127 (0.9719, 1.0552)	0.9620 (0.9120, 1.0147)	1.0078 (0.9838, 1.0324)	1.0097 (0.9653, 1.0562)	0.9715 (0.9362, 1.0081)	0.9961 (0.9828, 1.0096)
**Fossil fuels**	1.0084 (0.9683, 1.0503)	1.0537 (0.999, 1.1108)	1.0015 (0.9779, 1.0256)	0.9940 (0.9507, 1.0393)	1.0007 (0.9648, 1.0381)	0.9993 (0.9861, 1.0127)
**Passive smoking**	1.0259 (0.9826, 1.0711)	1.0010 (0.9465, 1.0586)	1.0159 (0.9905, 1.0419)	1.0432 (0.9951, 1.0937)	1.0253 (0.9862, 1.0659)	1.0086 (0.9945, 1.0230)

^† ^Odds ratio (95%CI) (Confidence interval) by 10 μg/m^3 ^increase of O_3_, SO_2_, NO_2 _and PM_10_, and 10°C increase in temperature in multilevel logistic models with slope as random effects, adjusted by height, BMI, sex and age; clustering by child

* p-value < 0.05

### Pulmonary Function

Students from school 1 presented a 3% deficit of FEV_1_%/FVC and 4% deficit for PEF comparing with students from school 2, but only during summer and fall. No difference in FEV_1 _was found between the two schools, during any season. More children from school 1 had abnormalities in lung function than in school 2 (17.9% vs.11.5%; p = 0.02). The principal alteration was the obstructive type, which frequency was greater in those students with greater exposure (10.4% vs. 5.3%; OR = 1.95, 95% CI 1.03-3.7), followed by the mixed pattern also more frequent in the same students (4.1% vs. 0.9%; OR = 4.69, 95% CI, 1.04-21.1).

In mixed models, after adjusting for height, BMI, sex, age, fossil fuel and passive smoking, results showed that NO_2 _levels were positively related to FVC, FEV_1 _and PEF, whereas O_3_, SO_2 _and PM_10 _were negatively related to FVC, FEV_1 _and PEF (Table 5).

**Table 5:** Association between lung functions with air pollutant levels and other exposures sources among infant residents in Salamanca, Guanajuato 2004 - 2005

	Lung function			
	FVC β^† ^(95%CI)	FEV_1 _β^† ^(95%CI)	PEF β^† ^(95%CI)	FEV_1_/FVC β^† ^(95%CI)
O_3 _(*μg/m*^3^)	-0.0746 (-0.1018, -0.0474)*	-0.0046 (-0.0064, -0028)*	-0.0160 (-0.0219, -0.0101)*	0.0578 (-0.0048, 0.1205)
SO_2 _(*μg/m*^3^)	-0.0248 (-0.0426, -0.0069)*	-0.0029 (-0.004, -0.0017)*	-0.0103 (-0.0142, -0.0064)*	-0.0278 (-0.069, 0.0133)
NO_2 _(*μg/m*^3^)	0.1572 (0.0648, 0.2496)*	0.0132 (0.0070, 0.0193)*	0.0484 (0.2840, 0.6869)*	0.0284 (-0.1845, 0.2414)
PM_10 _(*μg/m*^3^)	-0.0142 (-0.0274, -0.0009)*	-0.0028 (-0.0036, -0.0019)*	-0.1519 (-0.1807, -0.1230)*	-0.0691 (-0.0996, -0.0385)*
Temperature (°C)	0.3220 (0.0148, 0.4959)*	0.0263 (0.0147, 0.0379)*	1.1837 (0.805, 1.5623)*	0.03621 (-0.3635, 0.4359)
School	-0.3019 (-0.5400, -0.0638)*	-0.2800 (-0.1163, 0.0759)	-1.2844 (-1.8012, -0.7675)*	-1.8346 (-7.2676, 3.5982)
Atopy	-0.0252 (-0.1440, 0.0936)	-0.0201 (-0.1163, 0.0759)	0.0021 (-0.2371, 0.2415)	-0.4488 (-2.6628, 1.7640)
Allergen	0.0068 (-0.0550, 0.0686)	-0.0165 (-0.0665, 0.0335)	-0.0306 (-0.1552, 0.0939)	-0.4686 (-1.6205, 0.6832)
Fossil fuels	-0.0186 (-0.0798, 0.0425)	-0.0142 (-0.0637, 0.0352)	-0.1179 (-0.2412, 0.0052)	0.1946 (-0.9447, 1.339)
Passive smoking	-0.0219 (-0.0868, 0.0493)	-0.0276 (-0.0801, 0.0248)	-0.0966 (-0.2272, 0.0340)	-0.3535 (-1.5614, 0.8544)

^† ^Beta coefficients by 10 μg/m^3 ^increase of O_3_, SO_2_, NO_2 _and PM_10, _and 10°C increase in temperature, obtained with multilevel mixed models with slope as random effects, adjusted for height, BMI, sex, age, fossil fuel and passive smoking; clustering by child

* P-value < 0.05

Table 6 exhibits deficits in lung function (3-month rate) per IQR increase in air pollutant levels. After adjusting for potential confounding factors, results showed significant deficits (uni - pollutant models) in lung function associated with exposure to PM_10 _in boys and girls over the 1-year follow-up. An IQR increase in PM_10 _levels (IQR = 18.8 μg/m^3^) was significantly associated with 3-month deficits in FEV_1 _of -0.37% in girls and of -0.43% in boys, whereas deficits observed for PEF were -0.29% in girls and -0.36% in boys. Similar decreases were observed with bi-pollutant models.

**Table 6:** Effect of ambient air pollutants for the 3-month rate of change in lung function in children by gender, Salamanca, Guanajuato 2004 - 2005

	FVC (%)	**FEV**_**1 **_**(%)**	PEF (%)	**FEV**_**1**_**/FVC**
**Models**^**#**^	Percent change (95%CI)	Percent change (95%CI)	Percent Change (95%CI)	% (95%CI)
Girls				
*One-pollutant model*				
O_3 _8 hr-average ^&^	-0.21 (-0.84, 0.45)	-0.34 (-1.21, 0.57)	-0.27 (-1.55, 0.98)	0.24 (0.11, 0.37)*
PM_10 _24 hr-average ^§^	-0.23 (-1.26, 0.82)	-0.37 (-0.56, -0.13) *	-0.29 (-0.45, -0.14) *	0.11 (0.04, 0.18)*
SO_2 _24 hr-average ^¶^	-0.09 (-0.33, 0.18)	-0.11 (-0.61, 0.42)	-0.04 (-0.76, 0.65)	0.15 (0.03, 0.28)*
*Two-pollutant models*				
O_3 _8 hr-average ^&^	-0.12 (-0.46, 0.25)	-0.14 (-0.37, 0.07)	-0.11 (-0.45, 0.29)	0.19 (0.11, 0.27)*
PM_10 _24 hr-average ^§^	-0.23 (-0.75, 0.29)	-0.27 (-0.48, -0.06)*	-0.22 (-0.75, 0.31)	0.09 (-0.01, 0.19)
O_3 _8 hr-average ^&^	-0.18 (-0.62, 0.27)	-0.23 (-0.42, -0.03) *	-0.17 (-0.33, -0.02)*	0.23 (0.12, 0.34)*
SO_2 _24 hr-average ^¶^	-0.04 (-0.23, 0.14)	0.11 (-0.25, 0.45)	-0.06 (-0.47, 0.33)	0.10 (0.03, 0.18)*
PM_10 _24 hr-average ^§^	-0.37 (-0.69, -0.01) *	-0.13 (-0.44, 0.20)	-0.25 (-0.47, -0.03) *	0.05 (-0.03, 0.14)
SO_2 _24 hr-average ^¶^	-0.07 (-0.58, 0.44)	0.06 (-0.48, 0.61)	0.03 (-0.65, 0.69)	0.18 (0.10, 0.26)*
Boys				
*One-pollutant model*				
O_3 _8-hr average ^&^	-0.11 (-0.64, 0.42)	-0.36 (-0.61, -0.12)*	-0.22 (-0.66, 0.21)	0.21 (0.09, 0.33)*
PM_10 _24-hr average ^§^	-0.24 (-0.53, 0.07)	-0.43 (-0.76, -0.11)*	-0.36 (-0.68, -0.04)*	0.02 (-0.05, 0.13)
SO_2 _24 hr-average ^¶^	0.17 (-0.41, 0.76)	0.08 (-0.26, 0.42)	-0.05 (-0.37, 0.28)	0.13 (0.02, 0.24)*
*Two-pollutant models*				
O_3 _8 hr-average ^&^	-0.06 (-0.50, 0.38)	-0.15 (-0.43, 0.13)	-0.22 (-0.77, 0.34)	0.14 (0.05, 0.23)*
PM_10 _24 hr-average ^§^	-0.20 (-0.59, 0.21)	-0.66 (-1.08, -0.24)*	-0.57 (-1.01, -0.14) *	-0.003 (-0.05, 0.045)
O_3 _8 hr-average ^&^	-0.32 (-0.60, -0.04)*	-0.41 (-0.78, -0.05) *	-0.33 (-0.52, -0.15)*	0.16 (0.09, 0.24)*
SO_2 _24 hr-average ^¶^	0.14 (-0.43, 0.72)	0.09 (-0.31, 0.50)	-0.12 (-0.58, 0.34)	0.09 (0.02, 0.17)*
PM_10 _24 hr-average ^§^	-0.34 (-0.60, -0.10)*	-0.29 (-0.72, -0.15)*	-0.28 (-0.51, -0.06)*	0.03 (-0.08, 0.14)
SO_2 _24 hr-average ^¶^	0.05 (-0.61, 0.72)	0.06 (-0.63, 0.74)	0.03 (-0.87, 0.93)	0.14 (0.04, 0.25)*

*Definition of abbreviations: *CI = confidence interval; NO_2 _= nitrogen dioxide; O_3 _= ozone; PM_10 _= Particulate matter less than 10 μm in aerodynamic diameter

^# ^Mixed models adjusted for age, BMI, BMI square and residuals of the regression height on age. We used the log transformed version of the spirometric parameters

^&^% deficits in lung function, in milliliters per IQR (18.8) increase in 3-month mean pollutant concentration

^§ ^% deficits in lung function, in milliliters per IQR (50.60) increase in 3-month mean pollutant concentration

^¶ ^% deficits in lung function, in milliliters per IQR (6.40) increase in 3-month mean pollutant concentration

* p value < 0.05

Besides, we found that PM_10_, O_3 _and SO_2 _were positively associated with FEV_1_/FVC in boys and girls according to their changes per 3-month rate (Table 6).

## Discussion

It is well documented that high levels of many airborne pollutants can adversely affect many systems of the human body. In the present study, we found that pollutants in the city of Salamanca, Mexico were above the annual averages that specify the standards for air quality, so it could be considered that the inhabitants had chronic pollution exposure mainly from the thermoelectric plant, the oil refinery and the use of vehicles.

Air pollutants increased in winter in the school distant to major stationary air pollution sources, and at the same time wheezing, ARTI and dyspnea also increased in this school. ARTI and hospitalization secondary to this disease continued being more frequent in school 1, whereas dyspnea, eczema, wheezing and rhinorrhea were similar in both schools in winter. This could be explained at least in part by chronic exposure to air pollutants that has been previously related to infant bronchiolitis [9]; however, we did not find consistent effect or air pollution levels on symptom rates or lung function.

Spirometric abnormalities were more frequent in the school closer to the most polluted area. Pollutant levels were more often associated with "obstructive-type" than "restrictive-type" changes in lung function. However, there was a tendency for these levels to be negatively associated with FEV_1_, but positively associated with FEV_1_/FVC. Thus, our results do not present convincing evidence of an association of air pollution levels with obstructive lung function changes, or, for that matter, with restrictive lung function changes. Lung function has been one of the most important assessment tools available to investigators of the health effects of air pollution. It has been described that lung function in children is decreased on exposure to particulate matter air pollution (PM) and NO_2 _[10-12], also the estimated growth rate for children in the most polluted of the communities as compared with the least polluted has been predicted to result in a cumulative reduction of 3.4% in FEV_1 _and 5.0% in maximal midexpiratory flow over a 4-yr study period in a cohort of 3,035 southern California children [12]. Furthermore, the increase of endothelin-1 plasma levels, a potent vasoconstrictor that regulates pulmonary arterial pressure has been detected in higher levels in Mexican children exposed to chronic air pollution compared with controls [13]. In children from southwest Mexico City chronically exposed to ozone levels exceeding the U.S. National Ambient Air Quality Standards, showed bilateral hyperinflation, increased linear markings, and had a higher probability of developing interstitial markings with age [14]. The epidemiological implications of these findings are important for children residing in polluted environments, because bronchiolar disease could lead to chronic pulmonary disease later in life. In addition, evidence exists that O_3_, singly or in combination with SO_2_, produces oxidation when it comes into contact with pulmonary tissue and in this way acts as a powerful respiratory irritant that exacerbates the airway response to allergens [15-18] increasing respiratory symptoms.

In our study, there were positive and negative relationships between particular pollutants and respiratory symptoms along the year. This is difficult to explain, because each of these air pollutants can have different seasonal patterns and chemical interactions, the estimation and interpretation of each pollutant's individual risk estimates may not be straightforward. Multi-collinearity among the air pollution and weather variables also leaves the possibility of confounding and over- or under-fitting of meteorological and biological variables, thereby potentially influencing the health effect estimates for the various pollutants in differing ways. Thus, unmeasured characteristics related to school or environment such as natural allergens, other pollutants not evaluated, seasonal viral infections and/or genetic predisposition should be analysed. For instance, those subjects with atopy are more likely to have frequent asthma symptoms [19] and exposure to outdoor air pollutants could increase the risk of childhood asthma in these children [20].

In the students from school 1, the frequency of asthma was four times higher; rhinitis, nine times higher; and eczema, three times higher than reported for students living in Cuernavaca, Mexico where a lower level of air pollution has been reported [4]. The increase in allergens during spring and fall is a characteristic of the agricultural zone that surrounds this city, even more, the drop in temperature during the winter changes according to the direction and intensity of winds. Also the increase of contaminants in winter and seasonal viral infections could explain these results. For example, Rhinovirus infections are important triggers of acute asthma symptoms in susceptible persons such as those with allergic rhinitis and atopic asthmatic children experienced more frequent and severe virus-induced illnesses. Age and sex differences in the epidemiology of exacerbations remain less than fully explained, but hormonal and season influences are demonstrable [21,22].

PM_10 _levels were permanently above allowed levels during all year and even increased in winter. Also this was the most consistent factor related to FVC, FEV_1 _and PEF in boys and girls according to its changes per 3-month period. PMs have been associated with increased risk of pulmonary diseases and detrimental outcomes related to the cardiovascular system, including altered vessel functions, lung cancer, leukemia, lymphoma and central nervous system tumors [23]. They possess a great quantity of chemical compounds and, depending on their size, can lodge in the respiratory tract and even penetrate pulmonary tissue with long-term cumulative adverse effects on lung development in children from the age of 10 to 18 years, leading to clinically significant deficits in attained FEV_1 _as children reach adulthood [24].

Our study has some limitations such as we lacked a non-polluted control area and that sample size is small. Also, our results can't be generalized because of the particular environmental and geographic features in this city.

## Conclusions

Students from a school closer to the major stationary air pollution sources had in general more respiratory symptoms than those from a distant school. However, there was not a consistent effect of air pollution levels on symptom rates or lung function. PM_10 _levels were the most consistent factor related to FVC, FEV_1_, PEF and FEV_1_/FVC coefficient in boys and girls according to its changes per 3-month period.

## Competing interests

The authors declare that they have no competing interests.

## Authors' contributions

BL conceived the study, and participated in its design and coordination. JG made substantial contributions to conception and design and drafted the manuscript. NA was involved in drafting the manuscript and revising it critically for important intellectual content. VM, JP and RC participated in the analysis and interpretation of data. All authors read and approved the final version.

## Authors' information

JP and RC belong to the EMPECE Study Group.

## Pre-publication history

The pre-publication history for this paper can be accessed here:

http://www.biomedcentral.com/1471-2466/10/62/prepub
